# Spectrum of Morphologic Features of Lupus Nephritis According to Nephrology/Renal Pathology Society (ISN/RPS) Classification

**DOI:** 10.7759/cureus.10520

**Published:** 2020-09-18

**Authors:** Atif A Hashmi, Javaria Ali, Mansoor Rahman, Anab Rehan Taseer, Jasvindar Kumar, Muhammad Irfan

**Affiliations:** 1 Histopathology, Liaquat National Hospital and Medical College, Karachi, PAK; 2 Internal Medicine, Khyber Medical College, Peshawar, PAK; 3 Internal Medicine, Hayatabad Medical Complex, Peshawar, PAK; 4 Internal Medicine, Khyber Teaching Hospital, Peshawar, PAK; 5 Statistics, Liaquat National Hospital and Medical College, Karachi, PAK

**Keywords:** systemic lupus erythematosis, lupus nephritis, proteinuria, glomerulonephritis, secondary glomerulopathy, nephrology/renal pathology society (isn/rps) classification

## Abstract

Introduction

Lupus nephritis is one of the most important secondary glomerulopathy and a significant cause of chronic renal failure. Early diagnosis is key to improved prognosis. The International Society of Nephrology/Renal Pathology Society (ISN/RPS) classification stratifies renal biopsy evaluation in different classes that correlates with clinical renal outcome. In the current study, we aimed to evaluate the proportion of patients in each class of lupus nephritis in our population.

Methods

We evaluated renal biopsies of 128 patients that fulfilled the clinical and serologic criteria of lupus nephritis. The histologic classification was done according to the ISN/RPS classification, and immunofluorescence studies were performed. Active and chronic lesions were assessed on renal biopsy, and association of different histopatholgic parameters with lupus classes was done.

Results

The mean age was 28.85±12.24 years. Most of the patients were from age group ≤25 years (48.4%). Active lesions were seen in 66 (51.6%) patients, with endocapillary hypercellularity being the most common active lesion type, i.e. 81.8%. Chronic lesions were noted in 42 (32.8%) patients, with glomerular sclerosis being the most common chronic lesion type, i.e. 69%. Majority of the patients belonged to class IV (46.9%). Females were more likely to present at advanced lupus stage compared to males, and older patients (>50 years) had a higher chance to present at a late stage (class IV and higher). Active lesions were significantly found more frequently in classes III and IV, while chronic lesions were more likely to present in classes III to V.

Conclusion

We found that a significant proportion of patients of lupus nephritis in our population presents at an advanced stage as more than 60% patients were of class IV or higher. This signifies lack of appropriate clinical surveillance of patients and assessment of renal functions early in disease course. This also necessitates revision of our locoregional protocols to manage lupus nephritis patients and a need to perform a renal biopsy early in disease course.

## Introduction

Glomerular diseases are one of the most important insidious sources of chronic renal failure [1]. Although primary glomerulopathies remain an important cause [2], most of the cases result from secondary etiologies among which diabetes and systemic lupus erythematosis (SLE) are in top of the list. Renal involvement in SLE is sometimes not readily apparent leading to late presentation and poor outcome. Serologic screening with evaluation of renal parameters like serum creatinine is paramount in early identification of lupus nephritis (LN). Significantly impaired renal parameters like presence of proteinuria warrants further assessment via renal biopsy. Although the cutoff for proteinuria varies, low cutoff like 500 mg/day is proposed as it is well established that early diagnosis improves prognosis in LN [3,4].

The International Society of Nephrology/Renal Pathology Society (ISN/RPS) proposed a classification for LN that was periodically updated over a period of time [5,6]. Various studies validated the ISN/RPS classification and determined the burden of disease in each class in different populations; however, such studies are scarcely available in our population. Therefore, in the current study, we aimed to evaluate the proportion of patients in each class of LN in our population.

## Materials and methods

It was a cross-sectional retrospective study conducted at Liaquat National Hospital and Medical College, Department of Histopathology, and was carried out from January 2016 till December 2018 for a period of three years. Core renal biopsies from 128 patients fulfilling the histopathological and immunological criteria [7] for the diagnosis of LN were included. Those patients who did not have an adequate biopsy or did not satisfy the histopathological and immunological criteria, or any patient with a pathological disease resembling LN, but had never shown evidence of circulating dsDNA were excluded from the study.

**Figure 1:**
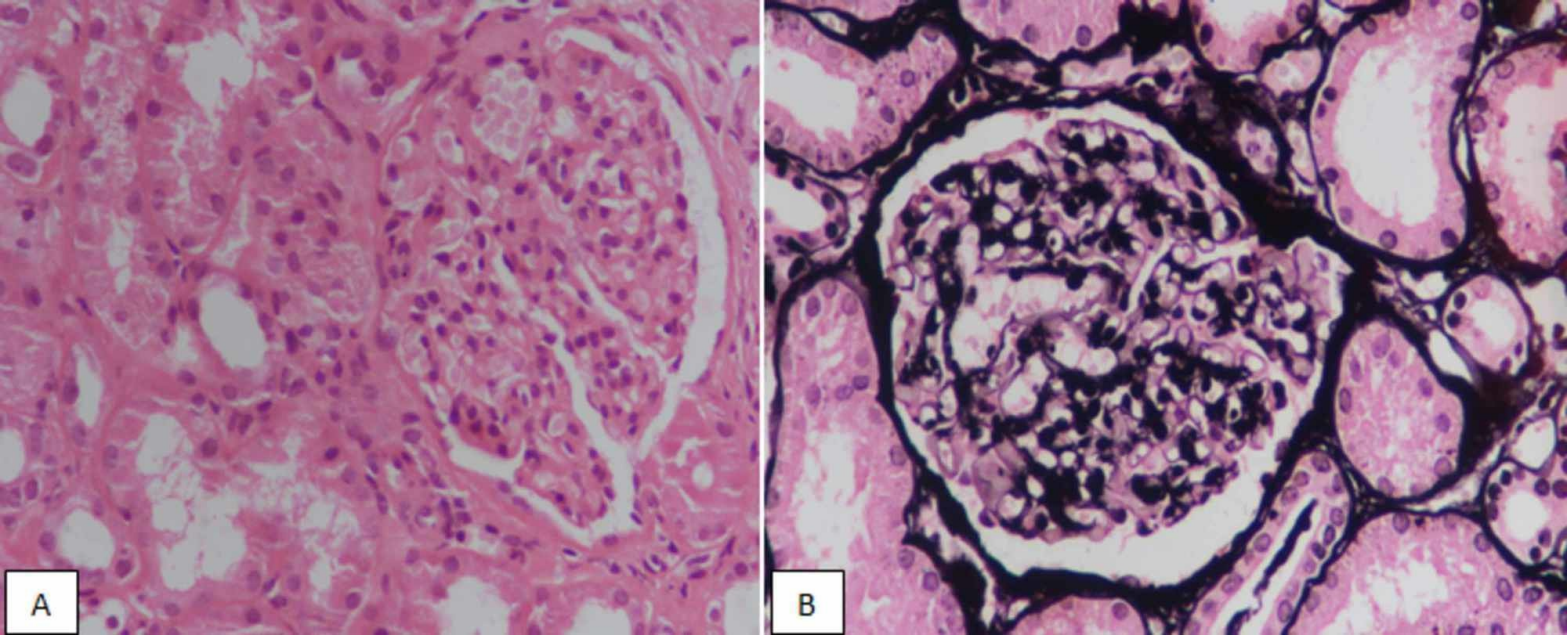
Class II lupus nephritis. (A) Hematoxylin and eosin staining at ×400 highlighting mesangial proliferation. (B) Silver staining demonstrating lack of segmental sclerosis or membrane thickening.

**Figure 2:**
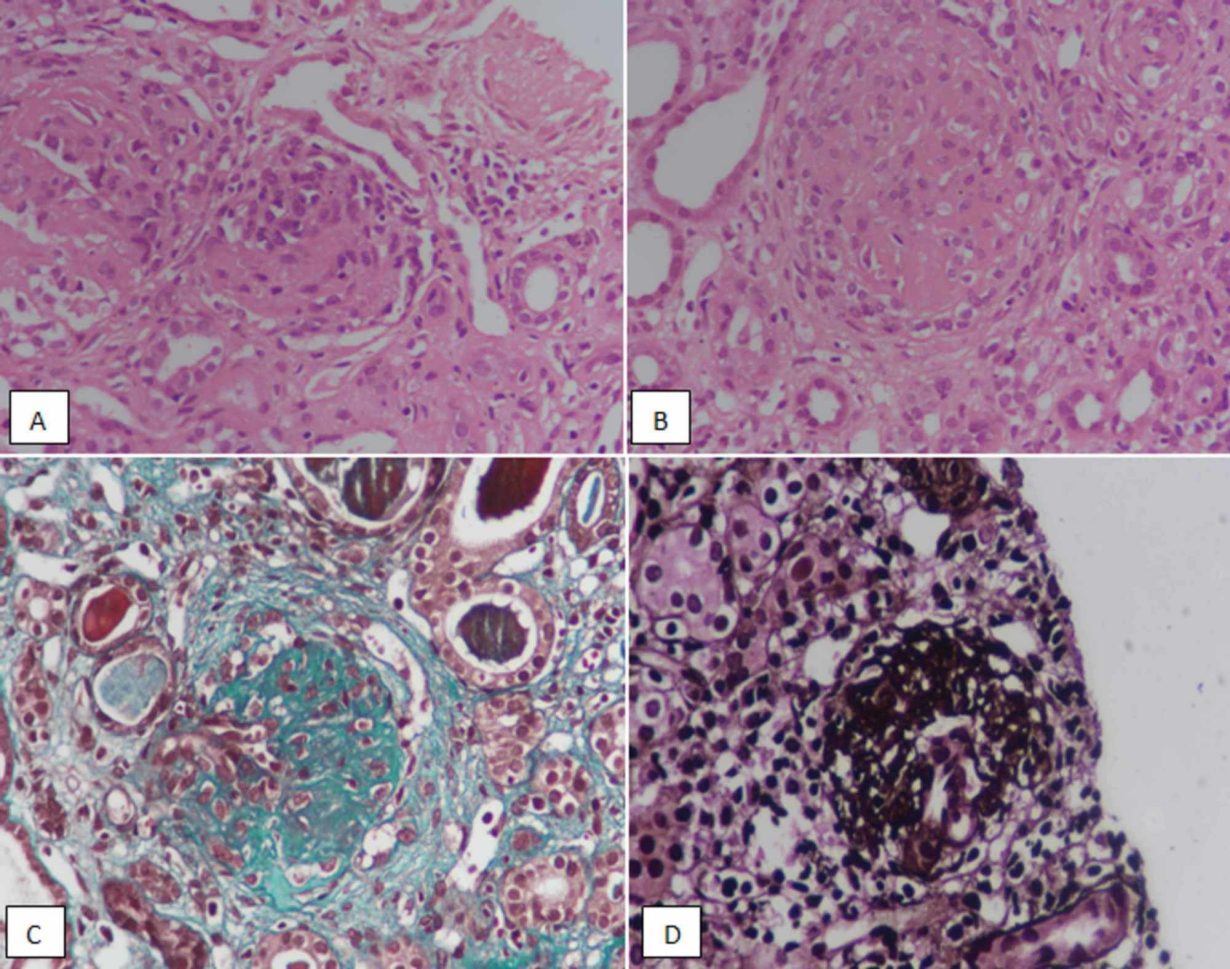
Class III lupus nephritis. (A, B) Hematoxylin and eosin-stained sections at ×400 demonstrating segmental sclerosing lesions in glomeruli. (C) Trichome stain highlighting segmental sclerosis. (D) Silver stain showing segmental sclerosis in a glomerulus.

**Figure 3:**
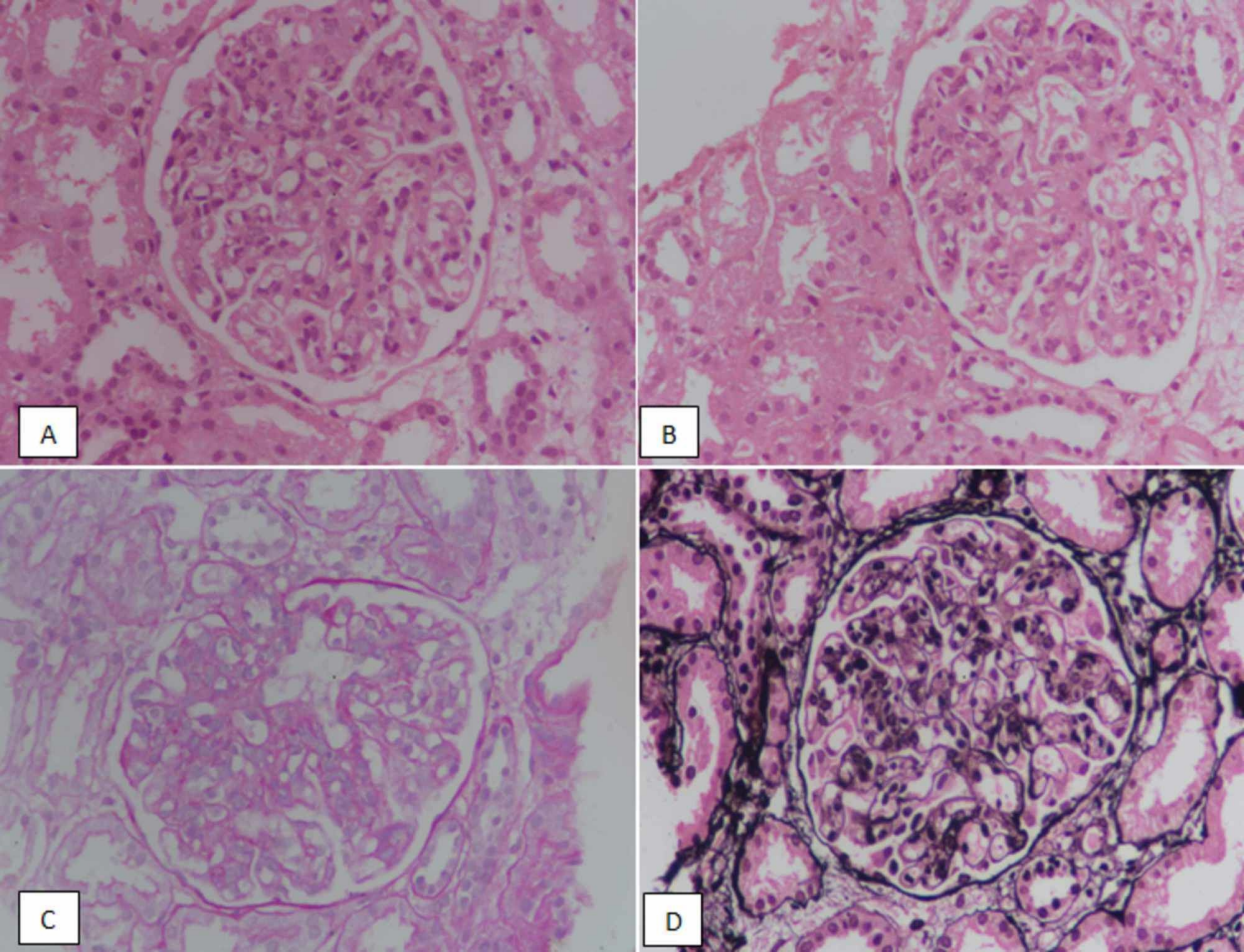
Class IV lupus nephritis. (A, B) Hematoxylin and eosin staining at ×400 highlighting endocapillary proliferation. (C) Periodic acid-schiff (PAS) staining demonstrating endocapillary proliferation and lack of membrane thickening. (D) Silver staining showing lack of membrane thickening.

**Figure 4:**
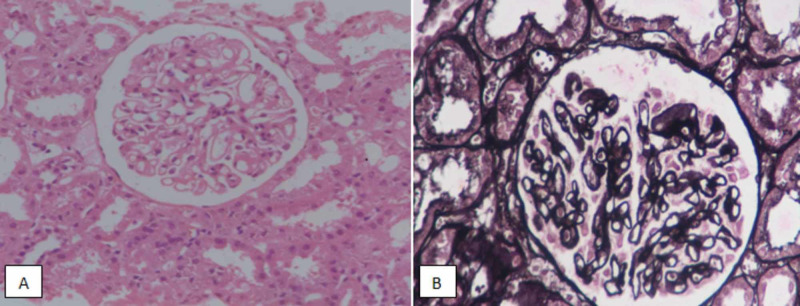
Class V lupus nephritis. (A) Hematoxylin and eosin staining at ×400 highlighting membrane thickening. (B) Silver staining demonstrating membrane thickening.

Biopsy specimens were processed for light and immunofluorescence (IMF) microscopy according to standard techniques in our center, including hematoxylin and eosin, periodic acid-Schiff, silver, and trichrome staining (Figures 1-4).

The histopathological classification of LN was done according to the ISN/RSP classification of LN from classes I to VI [8].

IMF scoring was interpreted by a senior consultant histopathologist on an intensity score of 0 to 3+, pattern was identified as linear or granular, and distribution of immune deposits was labeled as mesangial or membranous.

For data analysis, IBM SPSS Statistics for Windows, Version 20.0 (IBM, Armonk, NY) was used. Mean and standard deviation were evaluated for quantitative variables. Frequency and percentage were evaluated for qualitative variables. Chi-square test and Fisher's exact test were applied to determine association as appropriate. P-value ≤0.05 was considered significant.

## Results

**Figure 5:**
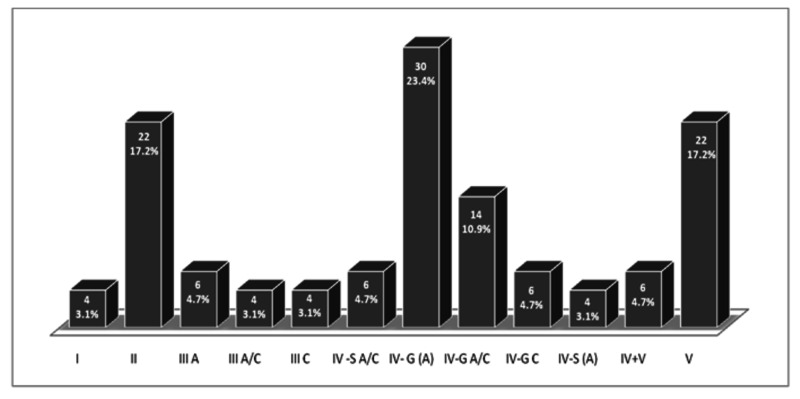
Distribution of patients into different classes according to the Nephrology/Renal Pathology Society (ISN/RPS) Classification.

Among 128 patients, 20 (15.6%) were male and 108 (84.4%) were female patients. The mean age was 28.85±12.24 years. Most of the patients were from age group ≤25 years (48.4%). Active lesions were seen in 66 (51.6%) patients, with endocapillary hypercellularity being the most common active lesion type, i.e. 81.8%. Chronic lesions were noted in 42 (32.8%) patients, with glomerular sclerosis being the most common chronic lesion type, i.e. 69%. Majority of the patients belonged to class IV (46.9%) (Table 1, Figure 5).

**Table 1:** Clinicopathologic characteristics of studied population

	Characteristic		Frequency (%)
Age (years)			
Mean±SD			28.85±12.24
Groups	≤25 years		62 (48.4)
	26-50 years		60 (46.9)
	>50 years		6 (4.7)
Gender	Male		20 (15.6)
	Female		108 (84.4)
Active lesions	Present		66 (51.6)
	Absent		62 (48.4)
Active lesions type (n=66)	Endocapillary hypercellularity		54 (81.8)
	Karyorrhexis		8 (12.1)
	Fibrinoid necrosis		4 (6.1)
Chronic lesions	Present		42 (32.8)
	Absent		86 (67.2)
Chronic lesions type (n=42)	Glomerular sclerosis		29 (69)
	Fibrous adhesions		9 (21)
	Fibrous crescents		4 (9.5)
Class	Class I		4 (3.1)
	Class II		22 (17.2)
	Class III		14 (10.9)
	Class IV		60 (46.9)
	Class V		22 (17.2)
	Class IV+V		6 (4.7)

Most of the cases showed full-house IMF pattern irrespective of class. Moreover, IgA and IgM were more frequently expressed in classes I, II, and V compared to classes III and IV (Table 2).

**Table 2:** Immunofluorescence pattern in different classes of lupus nephritis Fisher's exact test was applied *Significant as <0.05 **Not significant as >0.05

	n (%)						Total	P-Value
	Class I (n=4)	Class II (n=22)	Class III (n=14)	Class IV (n=60)	Class V (n=22)	Class IV+Class V (n=6)		
IgG	4 (100)	20 (90.9)	10 (71.4)	46 (76.7)	22 (100)	6 (100)	108 (84.4)	0.049*
IgM	4 (100)	22 (100)	14 (100)	56 (93.3)	22 (100)	6 (100)	124 (96.9)	0.456**
IgA	4 (100)	18 (81.8)	12 (85.7)	48 (80)	22 (100)	0 (0)	104 (81.3)	0.000*
C1q	4 (100)	22 (100)	14 (100)	22 (100)	6 (100)	6 (100)	128 (100)	N/A
C3c	4 (100)	20 (90.9)	12 (85.7)	56 (93.3)	22 (100)	6 (100)	120 (93.8)	0.550**

Females were more likely to present at advanced lupus stage compared to males and older patients (>50 years) had a higher chance to present at a late stage (class IV and higher). Active lesions were significantly found more frequently in classes III and IV, while chronic lesions were more likely to present in classes III to V (Table 3).

**Table 3:** Association of different clinicopathologic characteristics with lupus nephritis classes Fisher's exact test was applied *Significant as <0.05. **Not significant as >0.05

		n (%)						P-Value
		Class I (n=4)	Class II (n=22)	Class III (n=14)	Class IV (n=60)	Class V (n=22)	Class IV+Class V (n=6)	
Gender	Male	2 (50)	0 (0)	2 (14.3)	10 (16.7)	6 (27.3)	0 (0)	0.032*
	Female	2 (50)	22 (100)	12 (85.7)	50 (83.3)	16 (72.7)	6 (100)	
Age group	≤25 years	2 (50)	16 (72.7)	10 (71.4)	26 (43.3)	6 (27.3)	2 (33.3)	0.036*
	26-50 years	2 (50)	6 (27.3)	4 (28.6)	28 (46.7)	16 (72.7)	4 (66.7)	
	>50 years	0 (0)	0 (0)	0 (0)	6 (10)	0 (0)	0 (0)	
Active lesions	Present	0 (0)	0 (0)	10 (71.4)	54 (90)	0 (0)	2 (33.3)	<0.001*
	Absent	4 (100)	22 (100)	4 (28.6)	6 (10)	22 (100)	4 (66.7)	
Chronic lesions	Present	0 (0)	0 (0)	8 (57.1)	28 (46.7)	0 (0)	6 (100)	<0.001*
	Absent	4 (100)	22 (100)	6 (42.9)	32 (53.3)	22 (100)	0 (0)	

## Discussion

In the present study, we found that a significant proportion of patients of LN in our population presents at an advanced stage as more than 60% patients were of class IV or higher. Moreover, we found that active lesions are more likely to be present in classes III and IV compared to other classes.

Our results show that females were more likely to present at advanced lupus stage compared to males and older patients (>50 years) had a higher chance to present at a late stage (class IV and higher).

Active and chronic lesions are defined prognostic parameters in the ISN/RSP classification; we found that class III and IV patients were more likely to show active lesions on renal biopsy, while class III to V patients were more prone to have chronic lesions.

Various studies have validated the clinical importance of the ISN/RSP classification of LN. Studies have shown that, apart from classes I to VI, histological parameters like endocapillary hypercellularity, fibrinoid necrosis, and cellular crescents correlate with clinical renal outcome [9]. We found the presence of such active lesions in 51.6% of the cases.

Asian patients with SLE are shown to have higher frequency of kidney involvement compared to Western countries [10]. Moreover, more severe renal impairment was noted in Asian people suffering from LN [11]. A study conducted in Thailand, including 569 patients, found that 43.6% patients had nephrotic range proteinuria. Renal biopsy was done in 314 patients, among which 61.5% patients had a diffuse proliferative (class IV) pattern [12]. Although in our study we did not assessed frequency of renal involvement in SLE patients, we also found quite a high percentage of diffuse proliferative LN in our population, i.e. 46.9%, and 68.4% patients had IV or higher pattern (IV, V, and IV+V). Similarly, an Iranian study found an even higher frequency of class IV LN, i.e. 70.7% with 6.1% mortality in LN patients [13]. A multiethinic Asian study with more than 70% Chinese patients reported about two-thirds of LN patients with >class III pattern [14]. Demographic difference of frequency and severity of renal involvement of LN was also pointed out in a retrospective analysis [15]. This brief literature review clearly indicates grave disease severity in Asian LN patients and stipulates better management protocols and a need of early diagnosis.

Our study can be viewed with a few limitations. Most importantly, clinical follow-up of the patients was not available to correlate with histological findings, as high disease mortality was seen in other studies conducted in Asia. Therefore, we recommend more large-scale clinical studies to be done in our population to evaluate frequency of renal involvement in SLE patients and disease outcome/mortality in this subset of patients. 

## Conclusions

We found that a significantly higher proportion of our population with LN presents at an advanced stage according to the ISN/RSP LN classification. This signifies lack of appropriate clinical surveillance of patients and assessment of renal functions early in disease course and high threshold for performing a renal biopsy.
